# Regulation of Vascular Endothelial Growth Factor Signaling by Nicotine in a Manner Dependent on Acetylcholine-and/or β-Adrenergic-Receptors in Human Lung Cancer Cells

**DOI:** 10.3390/cancers15235500

**Published:** 2023-11-21

**Authors:** Hind Al Khashali, Ban Darweesh, Ravel Ray, Ben Haddad, Caroline Wozniak, Robert Ranzenberger, Stuti Goel, Jeneen Khalil, Jeffrey Guthrie, Deborah Heyl, Hedeel Guy Evans

**Affiliations:** Chemistry Department, Eastern Michigan University, Ypsilanti, MI 48197, USA; halkhash@emich.edu (H.A.K.); bdarwees@emich.edu (B.D.); rray9@emich.edu (R.R.); bhaddad1@emich.edu (B.H.); cwoznia5@emich.edu (C.W.); rranzenb@emich.edu (R.R.); sgoel1@emich.edu (S.G.); jkhalil2@emich.edu (J.K.); jguthri7@emich.edu (J.G.); dheylcle@emich.edu (D.H.)

**Keywords:** vascular endothelial growth factor, nicotine, lung cancer, nicotinic acetylcholine receptors, p53, PI3K, AKT, NFκB, β-adrenergic receptors, GABA

## Abstract

**Simple Summary:**

Nicotine, a highly addictive component in cigarette smoke, facilitates tumorigenesis and the accelerated development of non-small cell lung cancer (NSCLC), which is known to account for ~80% of all lung cancer cases. This study sheds light on how the nicotine treatment of NSCLC cells regulates vascular endothelial growth factor (VEGF) signaling, known to be important in the progression of vascular disease and cancer, by acting through nicotinic acetylcholine receptors and by leading to the activation of β-adrenergic receptors through increased levels of the stress neurotransmitters, norepinephrine/noradrenaline, and epinephrine/adrenaline. Nicotine-induced activation of VEGF promoted the function of proteins involved in increased cell survival and suppressed the function of a crucial tumor suppressor, blocking cell death. This work expands our scientific knowledge of mechanisms employed by nicotine in regulating VEGF signaling in a manner dependent on the acetylcholine and/or β-adrenergic receptors, leading to lung cancer cell survival, and also provides significant insights into novel future therapeutic strategies to combat lung cancer.

**Abstract:**

In addition to binding to nicotinic acetylcholine receptors (nAChRs), nicotine is known to regulate the β-adrenergic receptors (β-ARs) promoting oncogenic signaling. Using A549 (p53 wild-type) and H1299 (p53-null) lung cancer cells, we show that nicotine treatment led to: increased adrenaline/noradrenaline levels, an effect blocked by treatment with the α7nAChR inhibitor (α-BTX) but not by the β-blocker (propranolol) or the α4β2nAChR antagonist (DhβE); decreased GABA levels in A549 and H1299 cell media, an effect blocked by treatment with DhβE; increased VEGF levels and PI3K/AKT activities, an effect diminished by cell co-treatment with α-BTX, propranolol, and/or DhβE; and inhibited p53 activity in A549 cells, that was reversed, upon cell co-treatment with α-BTX, propranolol, and/or DhβE or by VEGF immunodepletion. VEGF levels increased upon cell treatment with nicotine, adrenaline/noradrenaline, and decreased with GABA treatment. On the other hand, the p53 activity decreased in A549 cells treated with nicotine, adrenaline/noradrenaline and increased upon cell incubation with GABA. Knockdown of p53 led to increased VEGF levels in the media of A549 cells. The addition of anti-VEGF antibodies to A549 and H1299 cells decreased cell viability and increased apoptosis; blocked the activities of PI3K, AKT, and NFκB in the absence or presence of nicotine; and resulted in increased p53 activation in A549 cells. We conclude that VEGF can be upregulated via α7nAChR and/or β-ARs and downregulated via GABA and/or p53 in response to the nicotine treatment of NSCLC cells.

## 1. Introduction

The number of deaths due to lung cancer is estimated to be ~350 per day, which is higher than that caused by pancreatic, breast, and prostate cancers combined [1,2]. Non-small cell lung cancer (NSCLC), composed largely of adenocarcinomas, is considered the most common type of lung cancer [3]. NSCLC accounts for 80–85% of all lung cancer cases and is considered to be highly resistant to existing cancer therapeutics [3].

Smoking is known to be a risk factor for NSCLC development and nicotine has been shown to promote growth and proliferation of cultured NSCLC cells [4,5,6,7,8]. Nicotine can affect many steps in cancer development and is able to activate mitogenic signaling pathways, increasing cell growth and survival [5,8,9]. Several reports have described nicotinic receptor-mediated pro-mitotic and anti-apoptotic signaling in a variety of cancer cells [7,8,9,10]. Nicotinic receptors composed of either α-subunits (homomeric nAChRs) or both α- and β-subunits (heteromeric nAChRs) have been identified in a variety of non-neuronal mammalian cells, regulating diverse cellular functions [7,8,9,10,11]. The activation of nAChRs occurs by binding of the endogenous ligand acetylcholine (ACh) or exogenous ligands such as nicotine [8,11,12]. The nAChRs have been reported to be present in human NSCLC cell lines that include A549 and H1299 used in this study [13,14]. Multiple nAChR subunits are known to be expressed in numerous non-neuronal cells; however, α7nAChR is thought to be the main receptor that facilitates nicotine-mediated cell growth [7,8,15,16]. While the most growth stimulatory nAChR is α7nAChR in cancer cells, the growth inhibitory receptor has been reported to be α4β2nAChR [10].

Lung cancer cells have been shown to express β-adrenergic receptors (β-ARs) that function to facilitate and promote cellular growth, proliferation, metastasis, and apoptosis resistance [17]. The binding of nicotine to α7nAChR was reported to stimulate colon cancer cells by increasing noradrenaline levels, indirectly activating β-AR signaling [18,19]. We previously found that cell viability and cisplatin resistance increased upon A549 and H1299 cell treatment with nicotine and/or brain-derived neurotrophic factor, while the opposite effects were observed upon treatment with the broad-spectrum β-AR antagonist, propranolol [20]. Acting via β-AR, adrenaline and noradrenaline have been shown to have strong stimulating effects on a range of cancer types, including lung cancer [17,21,22,23,24,25]. Using different cancer cell lines, adrenaline and noradrenaline have been shown to act via β-AR signaling, resulting in elevated secreted levels of vascular endothelial growth factor (VEGF), inducing tumor growth [26]. These effects were blocked by treatment with the β-AR antagonist, propranolol [26,27,28]. Cell treatment with the β1/2-AR agonist, isoproterenol, was found to result in the increased expression of matrix metalloproteinase-2/9 and VEGF, promoting A549 cell proliferation [29].

Nicotine treatment was reported to decrease production of the inhibitory neurotransmitter, γ-aminobutyric acid (GABA), and to increase the release of adrenaline and noradrenaline in lung cancer [23,30,31]. In addition, GABA treatment blocked the effects of nicotine stimulation and tumor development in mice [23,30,31].

In this study, we investigated a molecular mechanism underlying VEGF signaling by nicotine in two NSCLC cell lines, A549 (p53 wild-type) and H1299 (p53-null), and show that the nicotine treatment of NSCLC cells leads to increased levels of adrenaline, noradrenaline, VEGF, and activities of PI3K, AKT, NFκB, and also to decreased GABA levels and p53 activity via a mechanism involving α7nAChR, α4β2nAChR, and β-ARs, leading to cell survival.

## 2. Materials and Methods

### 2.1. Materials

Most of the materials used in this study were purchased as reported earlier [32,33,34,35,36,37,38]. Phosphate Buffered Saline (PBS), nitrocellulose membranes, nicotine, α-bungarotoxin (α-BTX), propranolol, epinephrine/adrenaline, norepinephrine/noradrenaline, and γ-aminobutyric acid were purchased from Sigma-Aldrich (Burlington, MA, USA). The BCA protein assay kit, caspase 3 (cleaved) colorimetric in-cell ELISA kit (62218), goat anti-rabbit IgG (H + L) secondary antibody (HRP, 31466), α-tubulin monoclonal antibody (DM1A), human IgG (hIgG) isotype control, SuperSignal west pico luminol (chemiluminescence) reagent, 3,3′,5,5′-tetramethylbenzidine (TMB), lipofectamine 2000 transfection reagent, and the halt protease and phosphatase inhibitor cocktail were purchased from ThermoFisher (Waltham, MA, USA). The human IGFBP-3 (EHIGFBP3, ThermoFisher) ELISA kit was used to quantitate the levels of IGFBP-3. Donkey anti-mouse IgG (HRP, ab205724) was from Abcam (Waltham, MA, USA). The human heparanase sandwich ELISA kit (ab256401) was used to quantitate the levels of heparanase. Dihydro-β-erythroidine hydrobromide (DhβE) was from Tocris. Human/primate VEGF antibody (MAB293-100) was purchased from R&D Systems. SignalSilence Control siRNA (Unconjugated, 6568), SignalSilence p53 siRNA I (6231), and p53 antibody (9282) were from Cell Signaling Technology.

### 2.2. Cell Culture

Human NSCLC cell lines, H1299 (ATCC CRL-5803) and A549 (ATCC CCL-185) were purchased from the American Type Culture Collection (ATCC, Manassas, VA, USA). Cells were cultured in DMEM/F12 media with 10% Fetalgro bovine growth serum, 50 U/mL streptomycin, 50 U/mL penicillin, in 25 cm^2^ tissue culture flasks and allowed to grow at 37 °C, 95% humidity, and 5% CO_2_ as we reported earlier [20,32,33,34,35,36,37,38,39,40]. The cells were counted using a hemocytometer after trypan blue staining.

### 2.3. Adrenaline, Noradrenaline, and GABA Concentration Determination

The concentrations of adrenaline (MyBioSource (San Diego, CA, USA), #MBS727241) and noradrenaline (MyBioSource, #MBS2602530) in the cell culture media were measured according to the manufacturer’s instructions. Briefly, a competitive enzyme immunoassay technique was used to quantitate adrenaline using an anti-adrenaline antibody and an adrenaline-HRP conjugate. The media were incubated together with the adrenaline-HRP conjugate in the pre-coated plate for one hour. The wells were then washed and incubated with the TMB substrate for HRP. After stopping the reaction, the color changed from blue to yellow. Following the development of the yellow color, the absorbance was measured at 450 nm using a microplate reader. Since adrenaline in the media and the adrenaline-HRP conjugate compete for the anti-adrenaline antibody binding site, the intensity of the color is inversely proportional to the adrenaline concentration. The concentration of noradrenaline was measured using a double antibody sandwich ELISA technique. The wells were precoated with an anti-human noradrenaline monoclonal antibody followed by detection using a biotinylated polyclonal antibody. The media and biotinylated antibodies were first added into ELISA plate wells. Following washing, avidin-peroxidase conjugates were added to the wells along with the TMB substrate. The color intensity and noradrenaline concentration in the media were positively correlated. The concentrations of GABA in the cell culture media were measured using the human GABA sandwich ELISA kit (ab287792) according to the manufacturer’s instructions. The color intensity was proportional to the amount of GABA captured from the media.

### 2.4. VEGF Concentration Determination

The concentration of VEGF in the cell culture media was measured using the human VEGF solid-phase sandwich ELISA kit (ThermoFisher, KHG0111) that measures the amount of VEGF bound between a matched antibody pair. The media were added to wells pre-coated with a target-specific capture antibody, then a second biotinylated detector antibody was added. The signal was measured following the addition of streptavidin-HRP and the substrate solution. The intensity of the signal was directly proportional to the VEGF concentration in the media.

### 2.5. p53 Transcription Factor Activity Assay

The p53 activity was assayed using the human p53 transcription factor activity assay kit (RayBio (Peachtree Corners, GA, USA), TFEH-p53), as we previously reported [37,38,41,42]. Briefly, double-stranded oligonucleotides, bound to 96-well plates that contained the p53 binding sequence, were used to quantitate active p53 in whole-cell lysates.

### 2.6. PI3K Assay

The PI3-kinase p85-alpha/gamma (Phospho-Tyr467/199) (Boster (Pleasanton, CA, USA), EKC2337) ELISA kit was used to assay the PI3K activity according to instructions provided by the manufacturer. For each treatment, the signals for phospho-PI3K and total-PI3K were each normalized to the cell number, quantitated using the crystal violet solution, and then the ratio of phospho-PI3K to total-PI3K was calculated and plotted.

### 2.7. AKT Assay

The AKT activity was quantitated using the AKT kinase activity ELISA kit (Abcam) according to instructions provided by the manufacturer, as we previously reported [20,36,37,38,40,41]. The assay uses a synthetic peptide as a specific substrate for AKT, and a polyclonal antibody that detects and binds the phosphorylated substrate.

### 2.8. NFκB Assay

The NFκB p65 (Phospho/Total) InstantOne sandwich ELISA kit (ThermoFisher) was used as we previously reported [37,40,41] according to instructions provided by the manufacturer. Signals for phospho (Ser536) and total NFκB were each normalized to the cell number, then the ratio of phospho (Ser536)/total NFκB was calculated and plotted for each treatment.

### 2.9. MTT Assay

The MTT reduction assay (Sigma-Aldrich) was used to measure cell viability in 96-well plates, as we previously reported [20,36,37,38,42]. Absorbance was measured at 570 nm in the linear range. Untreated cells were used as a positive control while wells containing only cell-free culture media and DMSO were used as negative controls. Statistical analysis was carried out with GraphPad Prism version 9.5.1 for Windows.

### 2.10. Apoptosis Assay

Activated (cleaved) caspase 3 and tubulin were measured simultaneously using the caspase 3 (cleaved) colorimetric assay in whole cells in triplicate (ThermoFisher), as we previously reported [43,44].

### 2.11. Immunodepletion

The conditioned media were immunodepleted (ID) according to our previously published reports [34,37,38,39,40,42] using anti-VEGF antibodies (20 μg/mL), and, as a negative control, human IgG isotype (hIgG, 20 μg/mL). The ID media were then carefully removed and analyzed for the presence of VEGF by ELISA (ThermoFisher) before use.

### 2.12. Western Blotting

Cell lysates were collected as indicated and analyzed according to our previous methods [20,32,35,36,37,38,40,41]. SuperSignal west pico luminol (chemiluminescence) reagent was used to develop the Western blots that were imaged using a Bio-Rad molecular imager. The same blot was stripped and reprobed using Restore Western Blot Stripping Buffer (ThermoFisher) according to instructions provided by the manufacturer. Bands were quantitated using Image J software (version 1.47). The original western blot figures can be found in Supplementary Materials File S1.

### 2.13. SiRNA Transfection

Transfections were carried out according to our previously reported methods [35,36,38,40,41,42]. Control siRNA or p53 siRNA (100 nM) were added to the cells. All data represent the mean ± S.D. of at least three–five independent experiments, each performed in triplicate.

### 2.14. Statistical Analysis

The analysis was performed as we previously reported [20,33,34,35,36,37,38,40,41]. An ordinary one-way analysis of variance (ANOVA) followed by Tukey’s post hoc multiple comparison test, were performed.

## 3. Results

### 3.1. Opposite Effects Were Observed on the Levels of Adrenaline, Noradrenaline and GABA in A549 and H1299 Cell Media upon Treatment with Nicotine

Earlier studies have demonstrated that nicotine could enhance proliferation in a range of in vitro cell culture models, an effect that could be blocked by nAChR antagonists, such as the α7 antagonist α-BTX, highlighting α7nAChR as a possible target for cancer therapy [16,45]. The stress hormone, adrenaline, was shown to be synthesized and secreted after nicotine stimulation in certain cancer cells, and nicotine was shown to stimulate the β-ARs, promoting a number of mitogenic and oncogenic signaling pathways [18,19,23]. Treatment of colon cancer cells with nicotine led to increased synthesis and the release of noradrenaline and adrenaline, an effect blocked by an α7nAChR antagonist [10,19]. Increased levels of the stress neurotransmitter noradrenaline were previously reported in nicotine-treated NSCLC cells, leading to increased cell proliferation that was reversed by cell treatment with either the α7nAChR antagonist (α-BTX), or the β-blocker (propranolol) [23].

**Figure 1:**
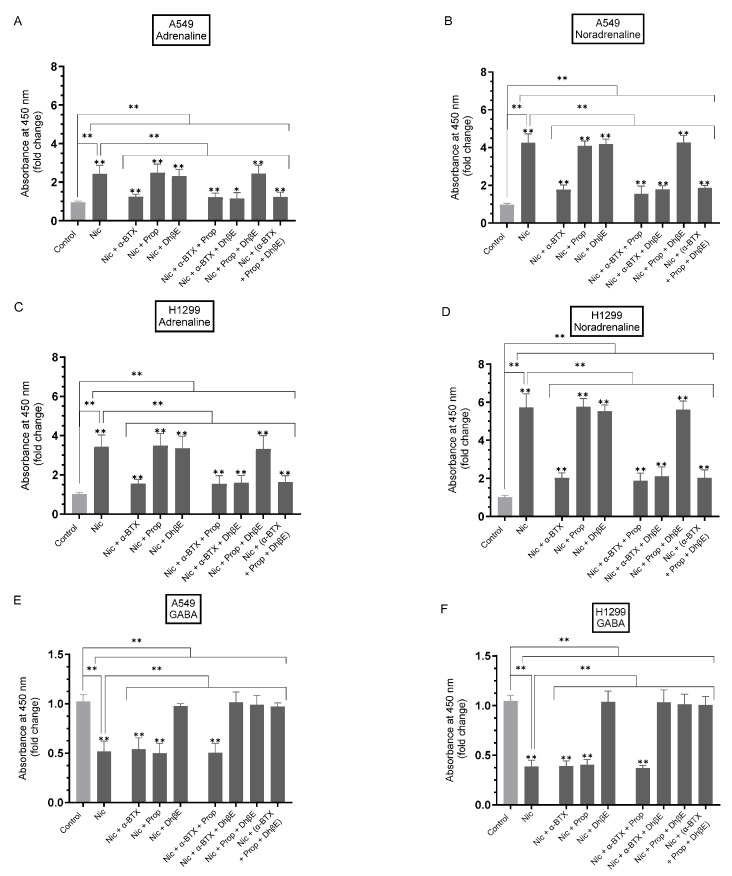
The levels of adrenaline and noradrenaline increased in the media of A549 and H1299 cells treated with nicotine, while opposite effects were observed on GABA levels. Cells (0.2 × 10^5^) were grown in 10% FBS-supplemented media for 24 h. The following day, the cell monolayers were serum-starved for 24 h, then incubated in serum-free media for 72 h in the absence or presence of nicotine (Nic, 1 µM), α-bungarotoxin (α-BTX, 200 nM), propranolol (Prop, 1 µM), DhβE (10 μM), or in combination. The concentrations of adrenaline (**A**,**C**), noradrenaline (**B**,**D**), and GABA (**E**,**F**) were measured as described in Section 2. Data were expressed as a fold change relative to the control untreated cells (Control) using the GraphPad 9.5.1 software (*n* = 5). Asterisks indicate a statistically significant difference from the corresponding control for each cell line. Statistical differences between different groups were analyzed by an ordinary one-way analysis of variance (ANOVA) followed by Tukey’s post hoc multiple comparison test, * *p* < 0.05, ** *p* < 0.01.

To examine the effect of cell treatment with nicotine on the levels of adrenaline/noradrenaline in A549 and H1299 cell media in the presence of inhibitors targeted against α7nAChR, β-AR, and/or α4β2nAChR, cells were grown in 10% FBS-supplemented media for 24 h (Figure 1). The following day, the cell monolayers were serum-starved for 24 h, then incubated in serum-free media for 72 h in the absence or presence of nicotine, the α7nAChR antagonist (α-BTX), the β-blocker (propranolol), the α4β2nAChR antagonist (DhβE), or in combination. The concentrations of adrenaline and noradrenaline were measured as described in Section 2.

The treatment of A549 cells with nicotine resulted in a ~2.45-fold increase in the levels of adrenaline (Figure 1A) and a ~4.25-fold increase in noradrenaline levels (Figure 1B) in the media. This increase in adrenaline/noradrenaline levels was blocked upon treatment of A549 cells with nicotine in the presence of α-BTX under all conditions tested (~2.0-fold decrease in adrenaline and ~2.45-fold decrease in noradrenaline compared to cells treated with only nicotine) but not upon addition of propranolol or DhβE (Figure 1A,B). Similar trends were found for H1299 cells under the same conditions (Figure 1C,D). H1299 cell treatment with nicotine increased the levels of adrenaline ~3.45-fold and noradrenaline ~5.70-fold (Figure 1C,D). Relative to cells treated with only nicotine, the addition of α-BTX to H1299 cells led to ~2.20-fold decrease in the levels of adrenaline and ~2.80-fold decrease in the levels of noradrenaline. These results suggest that treatment of A549 and H1299 cells with nicotine leads to enhanced levels of adrenaline/noradrenaline in the media in a manner dependent on α7nAChR but not on β-AR or α4β2nAChR.

GABA, known to be a suppressive neurotransmitter, has been shown to be synthesized and released by cancer cells regulating different functions in an autocrine manner [23,30,31]. GABA was shown to be secreted by A549 cells [46,47]. Treatment of NSCLC cells with nicotine led to downregulation of GABA synthesis, an effect reversed by cell co-treatment with the α4β2nAChR antagonist N-n-decylnicotinium iodide (NDNI), while cell treatment with GABA blocked β-AR signaling and cell proliferation [23].

To examine the effect of cell treatment with nicotine on the levels of GABA in A549 and H1299 cell media in the presence of inhibitors targeted against α7nAChR, β-AR, and/or α4β2nAChR, cells were grown in FBS-supplemented media for 24 h followed by serum starvation overnight. The cell monolayers were then incubated in serum-free media for 72 h in the absence or presence of nicotine, α-BTX, propranolol, and DhβE, or in combination (Figure 1E,F). The concentration of GABA was measured as described in Section 2. The treatment of cells with nicotine resulted in a ~2.0-fold decrease in the levels of GABA in the A549 cell media and ~2.50-fold decrease in the media of H1299 cells (Figure 1E,F). Relative to cells treated with only nicotine, no differences were found in GABA levels upon co-treatment of the cells with nicotine and either α-BTX and/or propranolol. The decrease in the levels of GABA observed with nicotine treatment, however, was reversed by co-treatment with DhβE alone or in combination with either α-BTX and/or propranolol (Figure 1). These results suggest the involvement of α4β2nAChR in modulating the levels of GABA in NSCLC cells.

### 3.2. Treatment with Nicotine Resulted in Increased VEGF Levels in A549 and H1299 Cell Media, an Effect Diminished by Cell Co-Treatment with α-BTX, Propranolol, and/or DhβE

Noradrenaline was reported to be important in promoting growth of gastrointestinal cancer and mediate nicotine signaling through VEGF activation [10,18,24,48]. Increased secreted levels of VEGF were observed in a variety of cancer cells in response to β-AR signaling activation by adrenaline and noradrenaline, leading to increased tumor growth [26,27,28]. Treatment with the β-AR antagonist, propranolol, blocked these effects [26,27,28]. Cell treatment with isoproterenol, the β1/2-AR agonist, was reported to enhance expression of VEGF increasing A549 cell proliferation [29].

**Figure 2:**
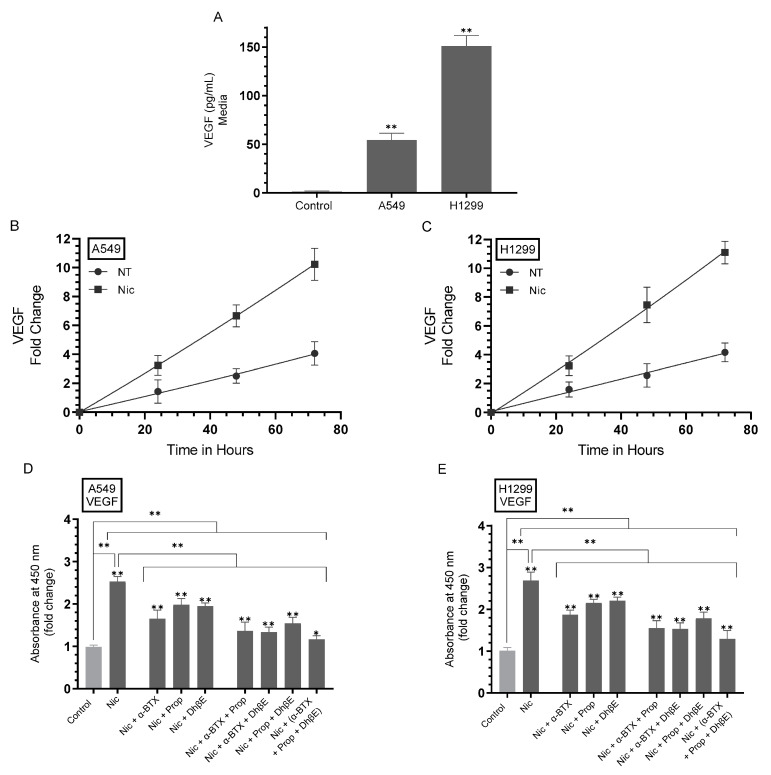
The levels of VEGF increased in the media of A549 and H1299 cells upon treatment with nicotine, an effect blocked by cell co-treatment with α-BTX, propranolol, and/or DhβE. Cells (0.2 × 10^5^) were grown in 10% FBS-supplemented media for 24 h. The following day, the cell monolayers were serum-starved for 24 h, the media replaced with fresh serum-free media (0 h) then the cells incubated for 72 h (**A**), and the concentration of VEGF was measured as described in Section 2. Control was media not incubated with cells. (**B**,**C**) The levels of VEGF were measured as a function of time without, or with, nicotine (Nic, 1 µM) and expressed as a fold change relative to 0 h. (**D**,**E**) The levels of VEGF in the presence of Nic without, or with, α-bungarotoxin (α-BTX, 200 nM), propranolol (Prop, 1 µM), DhβE (10 μM), or in combination were determined after 72 h of incubation. Data were averaged and expressed as a fold change relative to the control using the GraphPad 9.5.1 software (*n* = 5). Asterisks indicate a statistically significant difference from the corresponding controls while absence of asterisks indicates no significance. Statistical differences between different groups were analyzed by ANOVA followed by Tukey’s post hoc multiple comparison test, * *p* < 0.05, ** *p* < 0.01.

To examine the levels of VEGF in the media of nicotine-treated cells in the absence or presence of α-BTX, propranolol, and/or DhβE, cells were grown in 10% FBS-supplemented media for 24 h followed by serum starvation overnight. The media were then replaced with fresh serum-free media (0 h) then the cells incubated for 72 h (Figure 2A), and the concentration of VEGF was measured as described in Section 2. The levels of VEGF were measured as a function of time without, or with, nicotine and expressed as a fold change relative to 0 h (Figure 2B,C). The levels of VEGF in the presence of nicotine without, or with, α-BTX, propranolol, DhβE, or in combination were measured after 72 h of incubation (Figure 2D,E).

Previously, NSCLC cell lines were found to secrete higher levels of VEGF-A than normal human bronchial epithelial cells [49]. The levels of VEGF in H1299 cell media (~150 pg/mL) were higher than that found in the media of A549 cells (~55 pg/mL) (Figure 2A). Treatment of both cell lines with nicotine increased the levels of VEGF in A549 cell media ~2.50-fold and ~2.65-fold in H1299 cell media at 72 h post serum starvation (Figure 2B,C). Relative to treatment with only nicotine, co-treatment with the different inhibitors showed that α-BTX was most effective at reducing the levels of VEGF in the media of A549 cells (~1.50-fold decrease) as compared to cells co-treated with propranolol or DhβE (~1.25-fold decrease) (Figure 2D). A more pronounced decrease in VEGF levels was found upon A549 cell treatment with nicotine + α-BTX + propranolol (~1.80-fold decrease), nicotine + α-BTX + DhβE (~1.85-fold decrease), nicotine + propranolol + DhβE (~1.60-fold decrease), nicotine + α-BTX + propranolol + DhβE (~2.15-fold decrease) (Figure 2D). Comparable trends and fold reductions in the levels of VEGF in the media of H1299 cells were observed (Figure 2E). Relative to H1299 cell treatment with only nicotine, the levels of VEGF decreased ~1.40-fold upon co-treatment with α-BTX, ~1.20-fold upon co-treatment with either propranolol or DhβE, ~1.70-fold upon co-treatment with α-BTX + propranolol or with α-BTX + DhβE, ~1.50-fold upon co-treatment with propranolol + DhβE and ~2.00-fold with α-BTX + propranolol + DhβE (Figure 2E). These results suggest that nicotine regulates VEGF levels in the media via a mechanism involving α7nAChR, α4β2nAChR, and/or β-ARs.

### 3.3. Inhibition of p53 Activity upon A549 Cell Treatment with Nicotine Is in Part Reversed upon Cell Co-Treatment with α-BTX, Propranolol, and/or DhβE, or by Using Media Immunodepleted of VEGF—Moreover, VEGF Levels Increased in the Media upon A549 and H1299 Cell Treatment with Nicotine, Adrenaline, or Noradrenaline and Decreased by Cell Treatment with GABA While the Converse Was Found for the Activity of p53 in A549 Cells

Growing evidence suggests that p53 downregulates VEGF levels in NSCLC [27,50,51]. Mutant p53, that has lost its tumor suppressive activity, was reported to positively upregulate VEGF to promote tumor growth and cell survival [52]. Analysis of pan-cancer tissues demonstrated that mutated *TP53* serves as a marker for increased VEGF expression in cancer cohorts, including NSCLC adenocarcinoma [51].

The human NSCLC cell line, A549, used in this study, expresses relatively high levels of p53, while the human NSCLC cell line, H1299, is known to have a p53-null genotype due to a biallelic deletion of the *TP53* gene [53,54]. A549 cells were grown in media supplemented with 10% FBS for 24 h. The cells were then serum-starved overnight, then incubated in serum-free media for 72 h without, or with, nicotine, α-BTX, propranolol, DhβE, or in combination. The media were then collected and immunodepleted (ID) using either hIgG isotype control or anti-VEGF-specific antibodies (Section 2). The activity of p53 (Section 2) was measured by first seeding cells in 96-well plates in media supplemented with 10% FBS overnight. The cell monolayers were then incubated in serum-free media for 12 h, then treated with 300 μL (0.5 μg/μL) of the control or VEGF ID media for 72 h. The media containing the specific components in the various treatments were replaced every 12 h.

**Figure 3:**
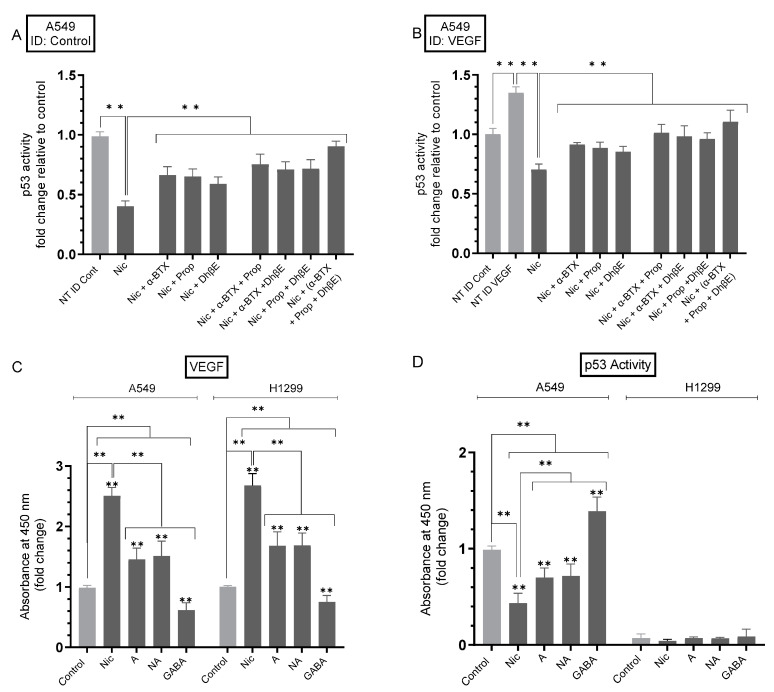
The activity of p53 is inhibited upon A549 cell treatment with nicotine, an effect partially reversed upon cell co-treatment with α-BTX, propranolol, and/or DhβE or by using media immunodepleted (ID) of VEGF. In addition, the levels of VEGF increased in the media of A549 and H1299 cells treated with nicotine, adrenaline, or noradrenaline and decreased by cell treatment with GABA while opposite effects were found on the p53 activity in A549 cells. Cells (0.2 × 10^5^) were grown in 10% FBS-supplemented media for 24 h. The following day, the cell monolayers were serum-starved for 24 h, then incubated in serum-free media for 72 h in the absence or presence of nicotine (Nic, 1 µM), α-bungarotoxin (α-BTX, 200 nM), propranolol (Prop, 1 µM), DhβE (10 μM), adrenaline (A, 10 nM), noradrenaline (NA, 10 nM), GABA (10 µM), or in combination. The activity of p53 was measured using cells incubated with media collected and ID using hIgG (20 μg/mL) as a control (**A**) or anti-VEGF-specific antibodies (**B**) (20 μg/mL) (Section 2). The concentration of VEGF (**C**) and the p53 activity (**D**) were measured (Section 2). Data were expressed as a fold change relative to cells not treated (NT) and ID with hIgG control (NT ID Cont) (**A**,**B**), control untreated cells (Control) of each cell line (**C**) or to A549 control (**D**) using the GraphPad 9.5.1 software (*n* = 5). Asterisks indicate a statistically significant difference while absence of asterisks indicates no significance. Statistical differences between different groups were analyzed by ANOVA followed by Tukey’s post hoc multiple comparison test, ** *p* < 0.01.

The activity of p53 increased ~1.35-fold upon ID of VEGF from A549 cell media as compared to media ID using hIgG control (Figure 3), suggesting that VEGF acts to suppress p53 activity in A549 cells. Cell treatment with nicotine led to ~2.5-fold decrease in p53 activity (Figure 3A). This decrease was more modest, ~1.40-fold, upon ID of VEGF from nicotine-treated cells (Figure 3B). A549 cells treated with nicotine and either α-BTX, propranolol, or DhβE resulted in ~1.65-fold higher p53 activity compared to cells treated with only nicotine (Figure 3A). The same treatments ID of VEGF led to ~1.20-fold increase in p53 activity upon co-treatment with nicotine and either α-BTX, propranolol, or DhβE compared to nicotine treated A549 cells (Figure 3B). A549 cell incubation with media prepared by treatment with (nicotine + α-BTX + propranolol), (nicotine + α-BTX + DhβE), (nicotine + propranolol + DhβE) and ID using hIgG antibody control resulted in ~1.85-fold increase in p53 activity relative to cells treated with nicotine (Figure 3A). That increase in p53 activity under the same conditions but using media ID of VEGF was ~1.35-fold (Figure 3B). Similarly, A549 cell incubation with media prepared by treatment with (nicotine + α-BTX + propranolol + DhβE) ID using hIgG control resulted in ~2.25-fold increase in p53 activity while that increase was ~1.55-fold upon ID of VEGF under the same conditions (Figure 3B). These results suggest that VEGF activation by nicotine treatment is important in regulating p53 activity and implicates the involvement of α7nAChR, α4β2nAChR, and β-ARs in the process.

Our results show (Figure 3) that VEGF is an important regulator of p53 activity in A549 cells untreated or treated with nicotine. To examine the effect of nicotine, adrenaline/noradrenaline, and GABA on the levels of VEGF and the p53 activity, cells were grown in 10% FBS-supplemented media for 24 h. The following day, the cell monolayers were serum-starved for 24 h, then incubated in serum-free media for 72 h in the absence or presence of nicotine, adrenaline, noradrenaline, or GABA at concentrations previously published to result in successful activation/inhibition [21,55,56,57,58]. The concentration of VEGF (Figure 3C) and the p53 activity (Figure 3D) were measured as described in Section 2.

Treatment with nicotine increased the levels of VEGF in the media of A549 cells by ~2.50-fold and ~2.65-fold in the media of H1299 cells (Figure 3C) relative to the control untreated cells. Treatment with adrenaline or noradrenaline led to ~1.45-fold increase and ~1.65-fold increase in the levels of the VEGF in the media of A549 and H1299 cells, respectively (Figure 3C). Conversely, VEGF levels decreased by ~1.65-fold in the media of A549 cells and by ~1.30-fold in H1299 cell media (Figure 3C) upon treatment with GABA. The opposite effects were found on p53 activation in A549 cells (Figure 3D). The activity of p53 decreased by ~2.30-fold with nicotine treatment and by ~1.40-fold with adrenaline/noradrenaline treatment and increased by ~1.40-fold upon A549 cell incubation with GABA (Figure 3D). Not surprisingly, there was no detection of p53 activation in H1299 cells since these cells are known to be p53-null [53].

### 3.4. The Levels of VEGF Increased in the Media of A549 Cells Untreated or Treated with Nicotine upon Knockdown of p53

**Figure 4:**
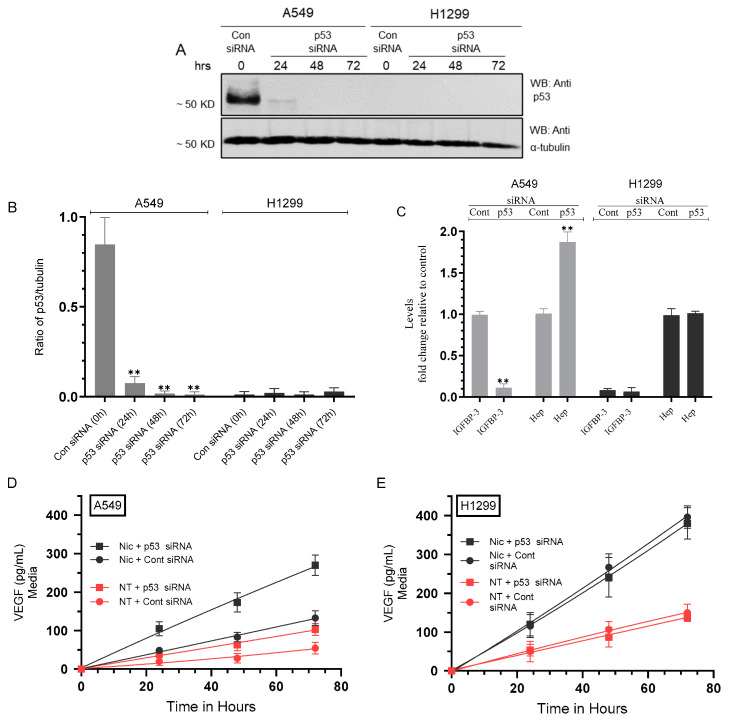
Knockdown of p53 increased the levels of VEGF in the media of A549 cells untreated or treated with nicotine. Cells (0.2 × 10^5^) were grown in media supplemented with 10% FBS overnight. The cell monolayers were then incubated in serum-free media for 24 h, then the media were replaced with fresh serum-free media (0 h). The cells were then incubated with the indicated siRNAs and either not treated (NT) or treated with nicotine (Nic, 1 µM) (Section 2). Western blotting (**A**) using the indicated antibodies was carried out on the same concentration of total protein (15 µL of 600 µg/mL) of the cell lysates. Quantitation of the Western blot, performed three times, was carried out using Image J 1.47 v software (**B**). To further verify p53 knockdown, the levels of IGFBP-3 and heparanase (Hep) were measured (**C**). The media were used to quantitate the levels of VEGF (Section 2, (**D**,**E**), *n* = 5), using the same concentration of protein (3 µL of 600 µg/mL total protein), as a function of time. Data were plotted using the GraphPad 9.5.1 software, ** *p* < 0.01.

Since A549 cell treatment with nicotine led to increased levels of VEGF in the media, which correlated with decreased p53 activation (Figure 3), we tested the effects of p53 knockdown on the levels of VEGF in the media of A549 cells without, or with, nicotine treatment (Figure 4). Cells were grown in FBS-supplemented media for 24 h then serum-starved overnight. The media were then replaced with fresh serum-free media (0 h). The cells were then treated with the indicated siRNAs (Figure 4), as described in Section 2 without, or with, nicotine. To further verify that knockdown of p53 was effective, the levels of IGFBP-3 and heparanase were measured (Figure 4A–C) since it is known that p53 binds directly to the heparanase gene promoter, blocking its transcription [59,60], and to induce IGFBP-3 expression [61]. We have also recently shown that knockdown of p53 led to enhanced heparanase levels and decreased IGFBP-3 levels in A549 cell media [38]. The same protein concentration of the media was used to quantitate the levels of VEGF as a function of time (Figure 4D,E) (Section 2). Compared to untreated cells transfected with control siRNA, nicotine treatment of A549 cells transfected with control siRNA led to ~2.50-fold increase in the levels of VEGF in the media (Figure 4) while that increase was ~2.70-fold in H1299 cell media (Figure 4). Relative to cells transfected with the control siRNA, the levels of VEGF in the media of A549 cells transfected with p53 siRNA increased by ~1.85-fold. Compared to untreated A549 cells transfected with p53 siRNA, incubation with nicotine increased VEGF levels by ~2.70-fold (Figure 4). As expected, no difference in VEGF levels was found in the media of H1299 cells transfected with control- or p53-siRNA since these cells are p53-null (Figure 4).

### 3.5. Treatment of A549 and H1299 Cells with Nicotine Led to Increased PI3K and AKT Activities, an Effect Diminished upon Cell Co-Treatment with Nicotine and α-BTX, Propranolol, and/or DhβE

**Figure 5:**
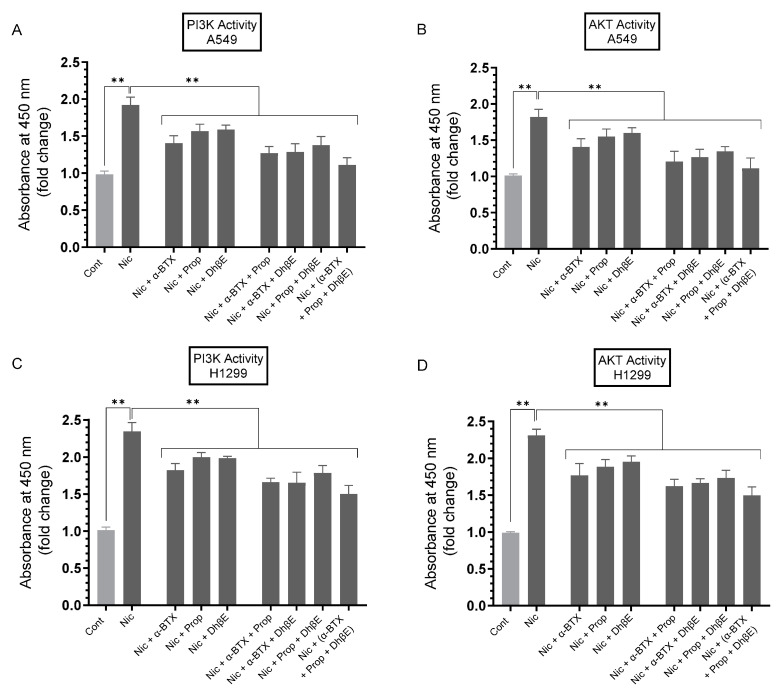
The activities of PI3K and AKT were increased upon A549 and H1299 cell treatment with nicotine (Nic) and decreased upon cell co-treatment with Nic and α-BTX, propranolol, and/or DhβE. Cells (0.2 × 10^5^) were grown in media supplemented with 10% FBS overnight. Cells were then serum-starved for 24 h, then incubated in serum-free media for 72 h in the absence or presence of nicotine (Nic, 1 µM), α-bungarotoxin (α-BTX, 200 nM), propranolol (Prop, 1 µM), DhβE (10 μM), or in combination. The PI3K activities (**A**,**C**) and AKT activities (**B**,**D**) were measured (Section 2). Data were expressed as a fold change relative to the control untreated cells (Control) using the GraphPad 9.5.1 software (*n* = 5). Statistical differences between different groups were analyzed by ANOVA followed by Tukey’s post hoc multiple comparison test, ** *p* < 0.01.

VEGF is known to stimulate PI3K/AKT activation, driving proliferation, lung tumor cell survival, growth, and metastasis [62,63]. To examine the effect of cell treatment with nicotine on the activities of PI3K and AKT in A549 and H1299 cells in the presence of inhibitors targeted against α7nAChR, β-AR, and/or α4β2nAChR, cells were grown in FBS-supplemented media for 24 h. The next day, the cell monolayers were serum-starved for 24 h, then incubated in serum-free media for 72 h in the absence or presence of nicotine, α-BTX, propranolol, DhβE, or in combination (Figure 5).

Treatment of A549 cells with nicotine increased the activities of PI3K and AKT ~1.90-fold and ~1.80-fold, respectively (Figure 5A,B). Relative to A549 cells treated with only nicotine, the fold decrease in the activity of PI3K upon co-treatment of A549 cells with nicotine and the inhibitors was as follows: (nicotine + α-BTX, ~1.35-fold), (nicotine + propranolol, ~1.25-fold), (nicotine + DhβE, ~1.20-fold), (nicotine + α-BTX + propranolol, ~1.50-fold), (nicotine + α-BTX + DhβE, ~1.50-fold), (nicotine + propranolol + DhβE, ~1.40-fold), (nicotine + α-BTX + propranolol + DhβE, ~1.70-fold) (Figure 5A). Comparable results were obtained for the AKT activity in A549 cells treated under the same conditions (Figure 5B). Treatment of H1299 cells with nicotine led to stimulation of the PI3K and AKT activities by ~2.35-fold and ~2.30-fold, respectively (Figure 5C,D). Relative to H1299 cells treated with only nicotine, the fold decrease in the activity of PI3K upon co-treatment of H1299 cells with nicotine and the inhibitors was as follows: (nicotine + α-BTX, ~1.30-fold), (nicotine + propranolol, ~1.15-fold), (nicotine + DhβE, ~1.15-fold), (nicotine + α-BTX + propranolol, ~1.40-fold), (nicotine + α-BTX + DhβE, ~1.40-fold), (nicotine + propranolol + DhβE, ~1.30-fold), (nicotine + α-BTX + propranolol + DhβE, ~1.55-fold) (Figure 5C). Comparable data were obtained for the AKT activity in H1299 cells treated under the same conditions (Figure 5D). These results suggest that treatment of A549 and H1299 cells with nicotine leads to PI3K/AKT activation in a manner dependent on α7nAChR, β-AR, and/or α4β2nAChR.

### 3.6. Addition of Anti-VEGF Antibodies to A549 and H1299 Cell Media Inhibited the Activities of PI3K, AKT, and NFκB in the Absence or Presence of Nicotine and Increased p53 Activation in A549 Cells

**Figure 6:**
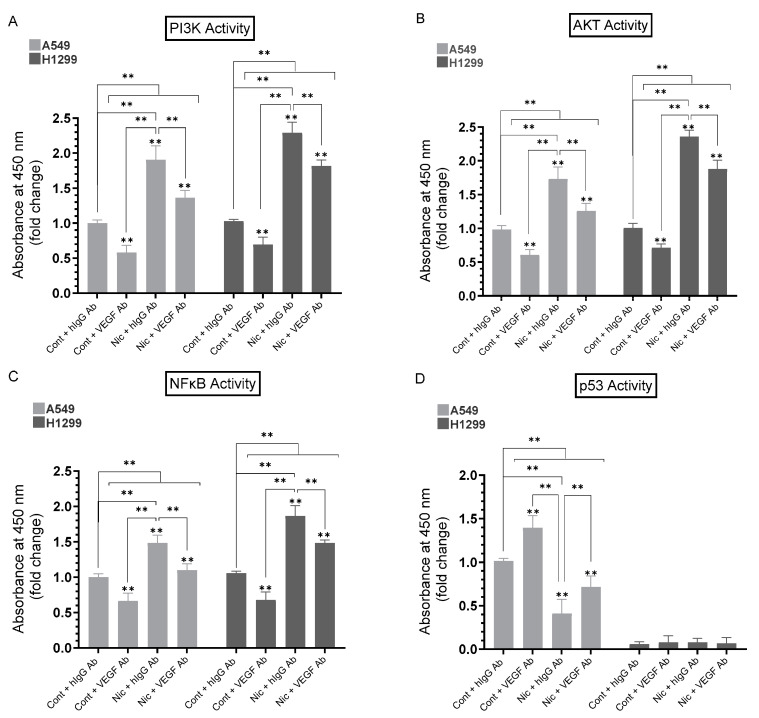
Anti-VEGF antibodies decreased the activities of PI3K, AKT, and NFκB in A549 and H1299 cells without or with nicotine and increased p53 activation in A549 cells. Cells were grown in media with 10% FBS for 24 h, serum-starved overnight, then incubated in serum-free media for 72 h without or with nicotine (Nic, 1 µM), hIgG (20 μg/mL) as a control, anti-VEGF-specific antibodies (20 μg/mL), or in combination. The activities of PI3K, AKT, NFκB, and p53 were measured (Section 2). Data were averaged and expressed as a fold change relative to the control cells treated with hIgG (Cont + hIgG Ab) of each cell line (**A**–**C**) or to A549 (Cont + hIgG Ab) (**D**) using the GraphPad 9.5.1 software (*n* = 5). Asterisks indicate a statistically significant difference from the control of each cell line. Statistical differences between different groups were analyzed by an ordinary one-way analysis of variance (ANOVA) followed by Tukey’s post hoc multiple comparison test, ** *p* < 0.01.

Our results (Figure 5) show that the treatment of A549 and H1299 cells with nicotine leads to PI3K/AKT activation. To examine the involvement of VEGF in nicotine-induced activation of PI3K/AKT, cells were grown in FBS-supplemented media for 24 h then serum-starved overnight. The cells were then incubated in serum-free media for 72 h in the absence or presence of nicotine, hIgG as a control, anti-VEGF-specific antibodies, or in combination (Figure 6). The activities of PI3K, AKT, NFκB, and p53 were then measured as described in Section 2.

Compared to cell incubation with hIgG control antibodies, the addition of VEGF antibodies to A549 cells led to a ~1.70-fold reduction in PI3K activity (Figure 6A), ~1.65-fold reduction in the activity of AKT (Figure 6B), ~1.50-fold reduction in NFκB activity (Figure 6C), and ~1.40-fold activation of p53 (Figure 6D). Relative to A549 cells treated with nicotine and incubated with control hIgG antibodies, the addition of VEGF antibodies to A549 cells treated with nicotine resulted in a ~1.35-fold reduction in PI3K, AKT, and NFκB activity (Figure 6A–C), and ~1.70-fold activation of p53 (Figure 6D).

Addition of VEGF antibodies to H1299 cells led to a ~1.45-fold reduction in PI3K activity (Figure 6A), ~1.40-fold reduction in the activity of AKT (Figure 6B), ~1.45-fold reduction in NFκB activity (Figure 6C) compared to cells incubated with hIgG control antibodies. As expected, there was no detectable p53 activity since H1299 cells are p53-negative. Relative to H1299 cells treated with nicotine and incubated with control hIgG antibodies, the addition of VEGF antibodies to H1299 cells treated with nicotine resulted in a ~1.25-fold reduction in PI3K, AKT, and NFκB activity (Figure 6A–C). These results clearly show that VEGF plays a role in regulating the activities of PI3K/AKT, NFκB, and p53. The levels of activity of PI3K, AKT, NFκB, and p53 under “Nic + VEGF Ab” conditions never returned to the level of “Cont + VEGF Ab” treatments, perhaps indicating the presence of a negative retro-control between VEGF and p53.

### 3.7. Treatment of A549 and H1299 Cells with Nicotine Led to Increased Cell Viability and Decreased Apoptosis, an Effect Blocked by Cell Co-Treatment with Nicotine and Either α-BTX, Propranolol, DhβE or in Combination—In Addition, Incubation of A549 and H1299 Cells with Anti-VEGF Antibodies Decreased Cell Viability and Increased Apoptosis without or with Nicotine Treatment

**Figure 7:**
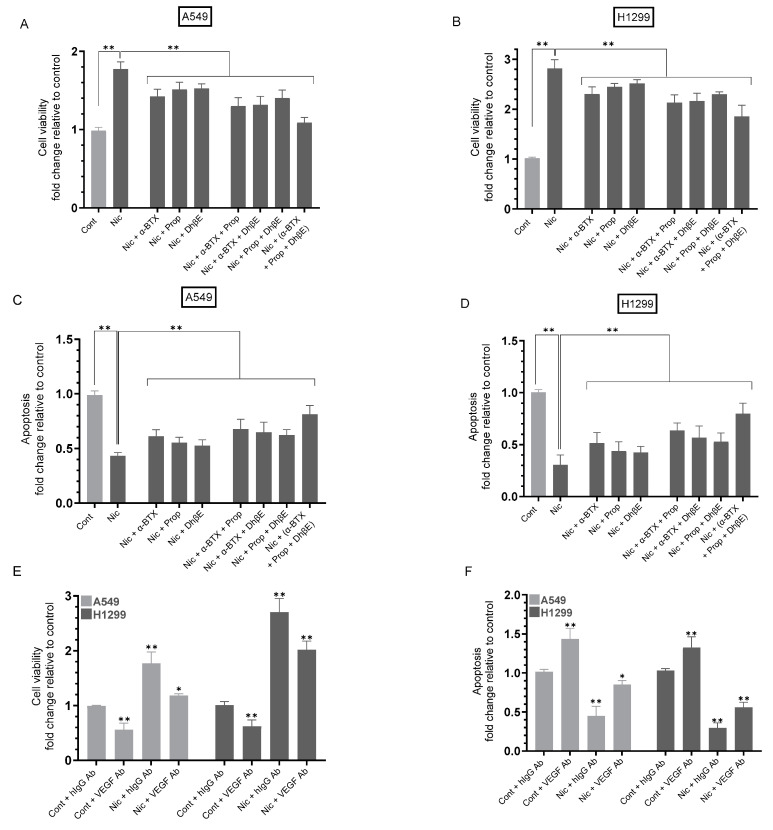
Compared to A549 and H1299 cell treatment with only nicotine (Nic), co-treatment with Nic and either α-BTX, propranolol, DhβE, or in combination resulted in decreased cell viability and increased apoptosis. In addition, anti-VEGF antibodies decreased cell viability and increased apoptosis of A549 and H1299 cells without, or with, nicotine treatment. Cells were grown in media with 10% FBS for 24 h, serum-starved overnight, then incubated in serum-free media for 72 h in the absence or presence of nicotine (Nic, 1 µM), α-bungarotoxin (α-BTX, 200 nM), propranolol (Prop, 1 µM), DhβE (10 μM), hIgG (20 μg/mL) as a control or anti-VEGF-specific antibodies (20 μg/mL), or in combination. Cell viability (**A**,**B**,**E**) and apoptosis (**C**,**D**,**F**) were measured (Section 2). Data were averaged, normalized, and expressed as a fold change relative to the control untreated cells (Cont) (**A**–**D**) or to control cells treated with hIgG (Cont + hIgG Ab) of each cell line (**E**,**F**) using the GraphPad 9.5.1 software (*n* = 5). Statistical differences between different groups were analyzed by an ordinary one-way analysis of variance (ANOVA) followed by Tukey’s post hoc multiple comparison test, * *p* < 0.05, ** *p* < 0.01.

In cancer cells, the most growth stimulatory nAChR is α7nAChR, whereas the growth inhibitory receptor is thought to be α4β2nAChR [10]. Like ACh, nicotine activates ionotropic cholinergic receptors such as α4β2 and α7 nAChRs [15,16,64]. To examine the effect of cell treatment with nicotine in the presence of inhibitors targeted against α7nAChR, β-AR, and/or α4β2nAChR on cell viability and apoptosis, A549 and H1299 cells were grown in FBS-supplemented media for 24 h. The next day, the cell monolayers were serum-starved for 24 h, then incubated in serum-free media for 72 h in the absence or presence of nicotine, α-BTX, propranolol, DhβE, or in combination (Figure 7). Cell viability (Figure 7A,B) and apoptosis (Figure 7C,D) were measured as described in Section 2.

Treatment with nicotine increased A549 cell viability ~1.75-fold (Figure 7A) and H1299 cell viability ~2.75-fold (Figure 7B). Relative to A549 cells treated with only nicotine, the fold decrease in cell viability upon co-treatment of A549 cells with nicotine and the inhibitors was as follows: (nicotine + α-BTX, ~1.25-fold), (nicotine + propranolol, ~1.20-fold), (nicotine + DhβE, ~1.15-fold), (nicotine + α-BTX + propranolol, ~1.35-fold), (nicotine + α-BTX + DhβE, ~1.35-fold), (nicotine + propranolol + DhβE, ~1.25-fold), (nicotine + α-BTX + propranolol + DhβE, ~1.60-fold) (Figure 5A). Relative to H1299 cells treated with only nicotine, the fold decrease in cell viability upon co-treatment of H1299 cells with nicotine and the inhibitors was as follows: (nicotine + α-BTX, ~1.20-fold), (nicotine + propranolol, ~1.15-fold), (nicotine + DhβE, ~1.10-fold), (nicotine + α-BTX + propranolol, ~1.30-fold), (nicotine + α-BTX + DhβE, ~1.25-fold), (nicotine + propranolol + DhβE, ~1.20-fold), (nicotine + α-BTX + propranolol + DhβE, ~1.50-fold) (Figure 7B). Apoptosis using the same treatments showed opposite trends for both cell lines (Figure 7C,D). Treatment with nicotine decreased the apoptosis of A549 cells ~2.30-fold (Figure 7C) and ~3.0-fold for H1299 cells (Figure 7D). Relative to A549 cells treated with only nicotine, the fold increase in apoptosis upon co-treatment of A549 cells with nicotine and the inhibitors was as follows: (nicotine + α-BTX, ~1.40-fold), (nicotine + propranolol, ~1.30-fold), (nicotine + DhβE, ~1.20-fold), (nicotine + α-BTX + propranolol, ~1.55-fold), (nicotine + α-BTX + DhβE, ~1.50-fold), (nicotine + propranolol + DhβE, ~1.45-fold), (nicotine + α-BTX + propranolol + DhβE, ~1.90-fold) (Figure 7C). Relative to H1299 cells treated with only nicotine, the fold increase in apoptosis upon co-treatment of H1299 cells with nicotine and the inhibitors was as follows: (nicotine + α-BTX, ~1.70-fold), (nicotine + propranolol, ~1.45-fold), (nicotine + DhβE, ~1.40-fold), (nicotine + α-BTX + propranolol, ~2.00-fold), (nicotine + α-BTX + DhβE, ~1.90-fold), (nicotine + propranolol + DhβE, ~1.75-fold), (nicotine + α-BTX + propranolol + DhβE, ~2.65-fold) (Figure 7D).

Our results (Figure 7) showed that nicotine treatment increased cell viability and decreased apoptosis in A549 and H1299 cells. Our results also showed higher levels of VEGF in the media of H1299 cells as compared to A549 cell media (Figure 2A) that were further increased upon treatment with nicotine (Figure 2B,C). Therefore, we next investigated the effect of neutralizing VEGF function using a VEGF-neutralizing antibody previously used in NSCLC [63]. Cells were grown in media supplemented with 10% FBS for 24 h, then serum-starved overnight. Cells were next incubated in serum-free media for 72 h in the absence or presence of nicotine, hIgG control, anti-VEGF-specific antibodies, or in combination (Figure 7). Cell viability and apoptosis were measured as described in Section 2. Data from five independent assays, each performed in triplicate, were averaged and expressed as a fold change relative to A549 or H1299 control cells treated with hIgG control using the GraphPad 9.5.1 software.

The addition of VEGF neutralizing antibodies to A549 cells decreased viability by ~1.80-fold (Figure 7E) and increased apoptosis by ~1.45-fold (Figure 7F) relative to cells incubated with hIgG antibodies. Similarly, incubation of H1299 cells with VEGF-neutralizing antibodies decreased cell viability by ~1.60-fold and increased apoptosis by ~1.30-fold (Figure 7) relative to cells incubated with hIgG antibodies. A549 cell treatment with nicotine increased cell viability by ~1.75-fold relative to the cells incubated with hIgG antibodies, an effect that was decreased by ~1.50-fold upon incubation with VEGF-neutralizing antibodies (Figure 7E). Relative to H1299 cells treated with nicotine, cell viability decreased by ~1.35-fold upon neutralization of VEGF function using VEGF neutralizing antibodies (Figure 7E). Treatment with VEGF-neutralizing antibodies of nicotine-treated cells increased A549 cell apoptosis ~1.85-fold and H1299 cell apoptosis ~1.90-fold (Figure 7F) relative to cells incubated with nicotine and hIgG antibodies.

## 4. Discussion

Cancer cells have been reported to express neurotransmitter receptors and synthesize a variety of neurotransmitters [31]. It is well established that nicotine exerts its biological action via binding to nAChRs; however, nicotine signaling has also been reported to trigger the production of β-AR ligands, such as adrenaline and noradrenaline, which in turn stimulate β-ARs, contributing to cell growth, proliferation, and the development of lung cancer [7,23]. Increased expression of many nAChRs, including α7nAChR, was detected in response to nicotine in human squamous cell carcinoma cells [65]. The high expression of α7nAChR levels in lung cancer cells was suggested to mediate nicotine-induced tumorigenesis [7,10,66]. In addition to the activation of nAChRs, nicotine was shown to promote cancer progression via activating β-AR, which frequently co-express with nAChRs on human lung cancer cells [7,18,20,23,31,42]. Treatment with nicotine was reported to increase mRNA expression of β1-, β2-AR in a dose-dependent manner in tumor tissues [7,19,23,31]. Nicotine was reported to induce adrenaline production and release, promoting colon cancer cell proliferation, effects that were reversed by using β-blockers [18,19,23,30,31]. Using oesophageal and gastric cancer cells, active components of cigarette smoke and stress hormones produced comparable results via β-ARs [22,31,67,68]. The treatment of NSCLC cells with nicotine increased the levels of the stress neurotransmitter noradrenaline, resulting in enhanced cell proliferation, an effect blocked by treatment with the α7nAChR antagonist α-BTX, or the non-selective β-blocker propranolol, which competitively antagonizes β-ARs [23]. The treatment of NSCLC A549 cells with propranolol was found to have antitumor activity, diminished cell survival, and increased apoptosis [69]. Recently, we reported that A549 and H1299 cell resistance to cisplatin increased with nicotine treatment while the opposite effects were observed upon cell treatment with propranolol [20]. In agreement with these reports, our results (Figure 1) show that treatment with nicotine led to increased adrenaline and noradrenaline levels in A549 and H1299 cell media. This increase was blocked by cell treatment with the α7nAChR inhibitor, α-BTX, but not upon the addition of either the β-blocker (propranolol), or the α4β2nAChR antagonist (DhβE) (Figure 1).

Previously, treatment with nicotine increased NSCLC cell proliferation while treatment with GABA, known to be a suppressive neurotransmitter, or the broad-spectrum β-AR antagonist, propranolol, had the opposite effect [23,30,31,47,58]. GABA has been reported to be synthesized and released by cancer cells, including A549 cells [23,30,31,46,47]. NSCLC cell treatment with GABA blocked β-AR signaling and cell proliferation while the exposure of NSCLC cells to nicotine resulted in the downregulation of GABA synthesis, an effect reversed by cell co-treatment with the α4β2nAChR antagonist NDNI [23]. Nicotine-induced stimulation of an autocrine noradrenaline-initiated signaling cascade along with GABA deficiency was suggested to contribute to the development of NSCLC in smokers [23,70]. In examining the effect of nicotine on the levels of GABA in A549 and H1299 cell media in the presence of inhibitors targeted against α7nAChR, β-AR, and/or α4β2nAChR, we found that treatment with nicotine resulted in decreased GABA levels in A549 and H1299 cell media, an effect blocked by cell co-treatment with the α4β2nAChR antagonist, DhβE, while no differences were observed in GABA levels upon co-treatment of the cells with nicotine and either α-BTX and/or propranolol (Figure 1). These results point to the involvement of α4β2nAChR in modulating the levels of GABA.

Accumulating evidence has shown a direct influence of the stress neurotransmitters adrenaline and noradrenaline on the migration, invasion, and promotion of a variety of tumor types, including lung cancer [17,29,30,69,71]. Stress has long been suspected to play a role in carcinogenesis; however, much remains to be learned about the underlying mechanisms [17,23,26]. A variety of cancer cells have been reported to have increased secreted levels of VEGF as a result of β-AR signaling activation by the stress-related mediators, adrenaline and noradrenaline, resulting in increased tumor growth [26,27,28]**.** Noradrenaline, adrenaline, and the nonspecific β-AR agonist, isoproterenol, were found to result in a significant increase in VEGF production by ovarian cancer cell lines, an effect blocked by cell treatment with the β1/2-AR antagonist, propranolol, indicating the involvement of β-AR in the process [28]. A549 cell treatment with isoproterenol was shown to lead to increased VEGF expression and enhanced cell proliferation [29]**.** Our results showed that the levels of VEGF in H1299 cell media were higher than that found in the media of A549 cells (Figure 2A), consistent with previous studies showing that NSCLC cell lines secreted higher levels of VEGF-A than normal human bronchial epithelial cells [49]. We also found that treatment with nicotine resulted in increased VEGF levels in A549 and H1299 cell media, an effect diminished by cell co-treatment with α-BTX, propranolol, and/or DhβE (Figure 2). These results suggest that nicotine regulates VEGF levels in the media of A549 and H1299 cells via a mechanism involving α7nAChR, α4β2nAChR, and/or β-ARs. Our findings also suggest that nicotine leads to increased adrenaline and noradrenaline levels (Figure 1) and a reduction in the levels of inhibitory GABA (Figure 1) that is concomitant with increased VEGF (Figure 2).

Mutant p53, devoid of its tumor suppressive function, was shown to positively upregulate VEGF to increase tumor cell growth and survival [52]. Several studies have reported that p53 downregulates the levels of VEGF in NSCLC [27,50,51]. Mutated *TP53* was shown to serve as a marker for the increased expression of VEGF in an analysis of pan-cancer tissues, including NSCLC adenocarcinoma [51]. The crosstalk between the p53 and VEGF pathways remains unclear. The VEGF promoter was shown to be directly repressed by p53 in endothelial cells [72]. Our data (Figure 3) show that the activity of p53 was inhibited upon treatment of A549 cells with nicotine. This inhibition was reversed, in part, upon A549 cell co-treatment with α-BTX, propranolol, and/or DhβE, or by the same cell treatments but using media immunodepleted of VEGF (Figure 3). These results indicate that the activation of VEGF resulting from nicotine treatment is important for the regulation of the activity of p53 and suggest the involvement of α7nAChR, α4β2nAChR, and β-ARs in the process. In addition, VEGF appears to be an important regulator of p53 activity in A549 cells untreated or treated with nicotine (Figure 3). The treatment of A549 and H1299 cells with nicotine, adrenaline, and noradrenaline resulted in increased VEGF levels in the media, while treatment with GABA led to a decrease in these levels (Figure 3C). The opposite effects were observed on the activity of p53 in A549 cells (Figure 3D). The activity of p53 decreased in A549 cells treated with nicotine, adrenaline/noradrenaline and increased upon A549 cell incubation with GABA (Figure 3D). Knockdown of p53 led to an increase in the levels of VEGF in the media of A549 cells untreated or treated with nicotine (Figure 4). No difference in VEGF levels was found in the media of H1299 cells transfected with control- or p53-siRNA since these cells are known to be p53-null (Figure 4). The activation of PI3K/AKT signaling is known to regulate several cellular processes necessary for tumorigenesis that include growth, proliferation and survival [73,74]. Earlier reports have provided a link between AKT and NFκB, in that AKT can regulate the transcriptional activity of NFκB [75,76,77]. The activity of NFκB has also been shown to be important for PI3K- and AKT-induced oncogenic transformation [76]. AKT-induced phosphorylation was found to result in NFκB translocation into the nucleus, enhancing transcription [75,76,77]. Inhibiting NFκB activity was also reported to decrease tumorigenicity [77]. Mutations of p53, common in lung adenocarcinoma, [78] are known to occur in ~35% of NSCLC patients [53,79,80,81]. Previous studies have shown that p53, known to negatively regulate transcription of the PI3K gene, suppresses EGFR/PI3K/AKT signaling by crosstalk with AKT through feedback loops to regulate NSCLC cell fate [79]. AKT was also found to confer resistance in NSCLC cells, in part via the downregulation of p53 [79]. Interactions between the PI3K/AKT and p53 pathways have been reported to occur via different mechanisms that include p53 regulation of the PI3K/AKT pathway by activation of the tumor suppressor, PTEN [82]. UV exposure of A549 cells resulted in increased expression of p53 and reduced levels of PI3K p110α and phosphorylated AKT [83]. The activation of Akt is known to block amplification of caspase activity via inactivating proapoptotic molecules [84,85,86] Previously, we reported that the increased activation of caspase-3 correlates with increased apoptosis and decreased NSCLC cell viability [20,35,36,37,38,40,41,42]. We previously found that the treatment of A549 cells with pifithrin-α increased the phospho/total NFκB ratio, suggesting that p53 is an antagonist of NFκB phosphorylation in A549 cells [40]. These results are consistent with previous reports showing that NFκB and p53 have opposing effects in cancer cells and an antagonistic signaling crosstalk [87]. NFκB activity increased in p53-null mice and p53 loss increased NFκB activation in a mouse model of KrasG12D-driven lung adenocarcinoma, while restoring p53 in p53-null lung tumors was found to inhibit NFκB, suppressing tumor growth [88,89]. PI3K/AKT activation is known to be increased by VEGF, enhancing cell proliferation and the progression of lung tumor cell survival and growth [62,63]. Our data showed that treatment of A549 and H1299 cells with nicotine resulted in increased PI3K and AKT activities, an effect that was diminished upon cell co-treatment with nicotine and α-BTX, propranolol, and/or DhβE (Figure 5), suggesting that nicotine leads to PI3K/AKT activation in a manner dependent on α7nAChR, β-AR, and/or α4β2nAChR. The addition of anti-VEGF antibodies to the media of A549 and H1299 cells blocked the activities of PI3K, AKT, and NFκB in the absence or presence of nicotine and resulted in increased p53 activation in A549 cells only (Figure 6). These results clearly show that VEGF plays a role in regulating the activities of PI3K/AKT, NFκB, and p53.

**Figure 8:**
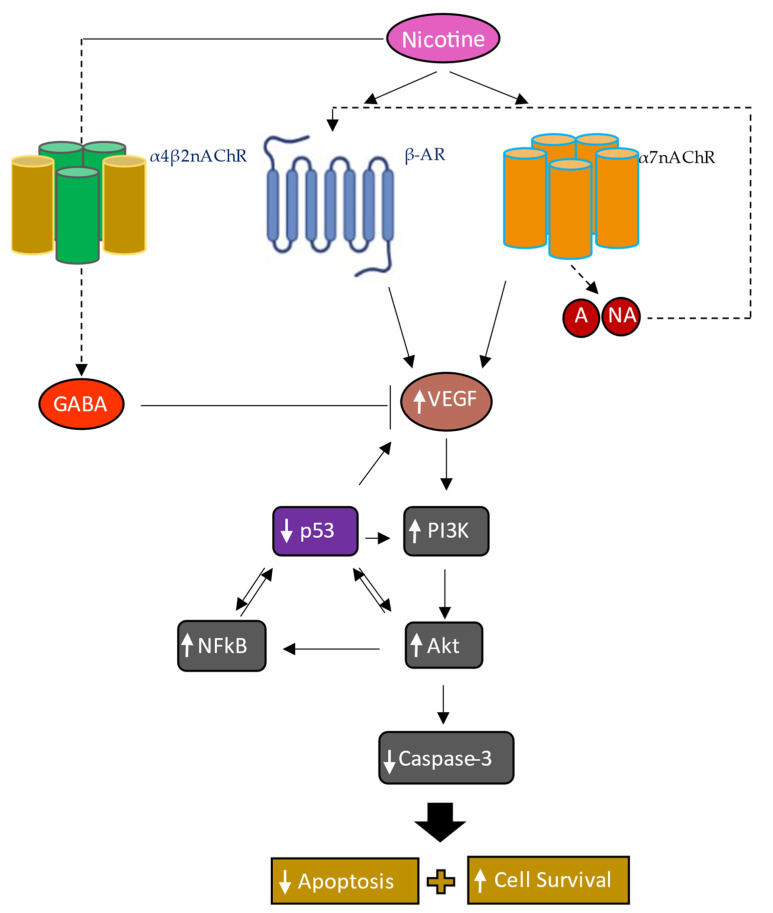
Summary of the main findings of this study. Nicotine enhances VEGF signaling in NSCLC by acting positively via the α7nAChR and β-ARs and negatively regulating GABA levels. Nicotine treatment leads to increased levels of adrenaline (A), noradrenaline (NA), VEGF, and activities of PI3K, AKT, NFκB, and decreased p53 activity via a mechanism involving α7nAChR, α4β2nAChR, and β-ARs leading to increased cell survival and decreased apoptosis. Dashed lines indicate a potential indirect effect. Colored boxes indicate new mechanisms provided from this study in NSCLC cells while gray boxes show findings from previous studies, from our laboratory and others, repeated here in this study under the same conditions to provide the overall scheme. While nicotine treatment led to increased levels of A/NA and decreased GABA levels in A549 and H1299 cell media, further evidence is needed to establish the direct involvement of the α7nAChR and α4β2nAChR in the regulation of these levels. Similarly, further experiments are needed to examine whether p53 acts directly on VEGF and vice versa.

In examining the effect of cell treatment with nicotine in the presence of inhibitors targeted against α7nAChR, β-AR, and/or α4β2nAChR on cell viability and apoptosis, we found that treatment of A549 and H1299 cells with nicotine enhanced cell viability and inhibited apoptosis, an effect blocked by cell co-treatment with nicotine and either α-BTX, propranolol, DhβE, or in combination (Figure 7). The addition of anti-VEGF antibodies to A549 and H1299 cell media decreased cell viability and increased apoptosis without, or with, nicotine treatment (Figure 7). The results presented in this manuscript (Figure 8) clearly show that there is a negative retro-control between VEGF and p53, as the knock-down of p53 led to an increase in the levels of VEGF while the blockade of VEGF led to an increase in p53 activity. Further experiments are needed to examine whether this retro-control is direct or indirect through PI3K/AKT.

## 5. Conclusions

In this study (Figure 8), we show that nicotine increases VEGF signaling in NSCLC by acting positively via the α7nAChR and β-ARs and by negatively regulating the levels of GABA. The treatment of NSCLC cells with nicotine resulted in increased levels of adrenaline, noradrenaline, VEGF, and activities of PI3K, AKT, NFκB, and also inhibited p53 activity via a mechanism involving α7nAChR, α4β2nAChR, and β-ARs, leading to decreased apoptosis and increased cell survival.

Novel findings from this study include: (1) the effect of cell treatment with nicotine on the levels of adrenaline/noradrenaline and GABA in A549 and H1299 cell media in the presence of inhibitors targeted against α7nAChR, β-AR, and/or α4β2nAChR; (2) changes in the levels of VEGF in the media of nicotine-treated cells in the absence or presence of α-BTX, propranolol, and/or DhβE, providing support for regulation of VEGF levels in the media of NSCLC cells by nicotine via a mechanism involving α7nAChR, α4β2nAChR, and/or β-ARs; (3) inhibition of p53 activity upon A549 cell treatment with nicotine is in part reversed upon cell co-treatment with α-BTX, propranolol, and/or DhβE, or by using media immunodepleted of VEGF, suggesting that VEGF activation by nicotine treatment is important in regulating p53 activity and implicates the involvement of α7nAChR, α4β2nAChR, and β-ARs in the process.; (4) VEGF acts to suppress p53 activity in A549 cells, however, no information can be provided as to whether this is a direct or indirect effect; (5) VEGF levels increased in the media upon A549 and H1299 cell treatment with nicotine, adrenaline, or noradrenaline and decreased by cell treatment with GABA, while the converse was found for the activity of p53 in A549 cells; (6) treatment of A549 and H1299 cells with nicotine led to increased PI3K and AKT activities, an effect diminished upon cell co-treatment with nicotine and α-BTX, propranolol, or DhβE; (7) the addition of anti-VEGF antibodies to A549 and H1299 cell media inhibited the activities of PI3K, AKT, and NFκB in the absence or presence of nicotine and increased p53 activation in A549 cells, indicating that VEGF plays a role in regulating the activities of PI3K/AKT, NFκB, and p53; (8) treatment of A549 and H1299 cells with nicotine led to increased cell viability and decreased apoptosis, an effect blocked by cell co-treatment with nicotine and either α-BTX, propranolol, DhβE or in combination; and (9) the incubation of A549 and H1299 cells with anti-VEGF antibodies decreased cell viability and increased apoptosis without, or with, nicotine treatment.

## Data Availability

The data presented in this study is available in this article.

## Supplementary Materials

The following supporting information can be downloaded at: https://www.mdpi.com/article/10.3390/cancers15235500/s1, File S1: The original western blot figures.
