# Supplementary material for: Regulation of Vascular Endothelial Growth Factor Signaling by Nicotine in a Manner Dependent on Acetylcholine-and/or β-Adrenergic-Receptors in Human Lung Cancer Cells
Source: Cancers (Basel). 2023 Nov 21;15(23):5500. doi: 10.3390/cancers15235500 (PMC10705358; doi:10.3390/cancers15235500)
Supplement: Supplementary file 1 [file cancers-15-05500-s001.zip › cancers-2640648-supplementary.pdf]

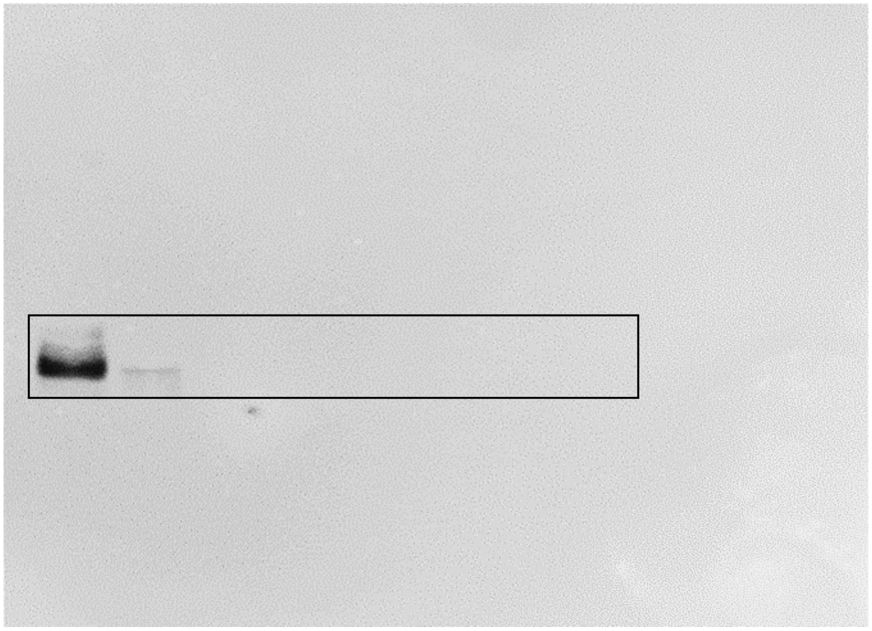

The image above was cropped to produce the image below.

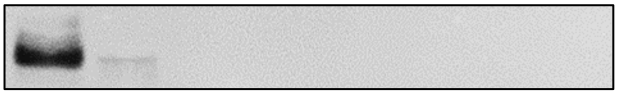

Shown on Figure 4A Top

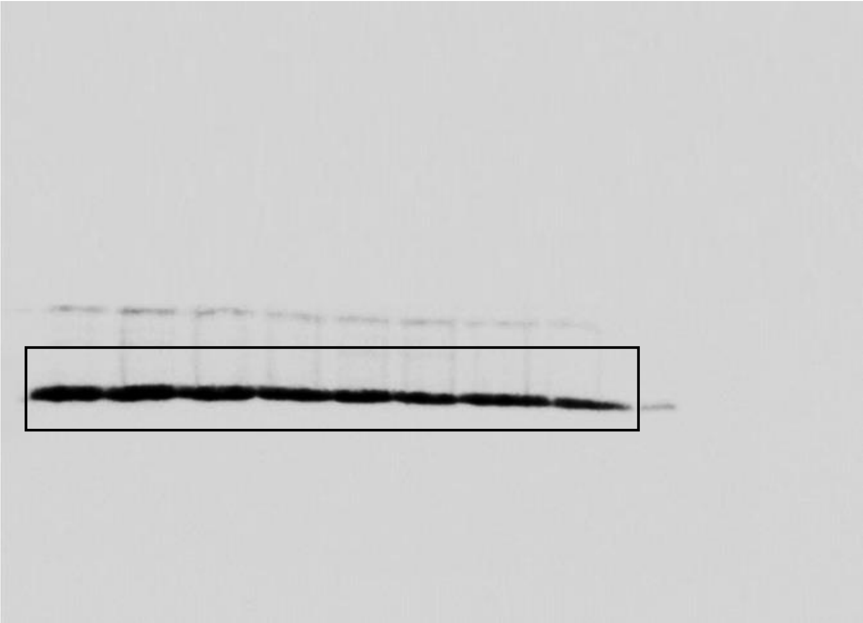

The image above was cropped to produce the image below.

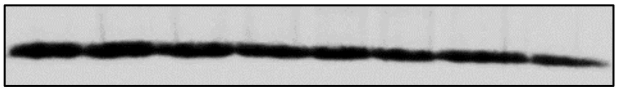

Shown on Figure 4A Bottom

File S1: The original western blot figures.
